# Leveraging State-Wide Partnerships to Develop a CNA Apprenticeship Program

**DOI:** 10.1093/geroni/igaf122.1014

**Published:** 2025-12-31

**Authors:** Anna Faul, Sam Cotton, Pamela Yankeelov, Barbara Gordon

**Affiliations:** University of Louisville, Louisville, Kentucky, United States; University of Louisville, Louisville, Kentucky, United States; University of Louisville, Louisville, Kentucky, United States; University of Louisville, Louisville, Kentucky, United States

## Abstract

Since 2015, our Geriatric Workforce Education Program (GWEP) has been committed to enhancing the skills of Certified Nursing Assistants (CNAs) across Kentucky by providing specialized training in dementia care best practices. Recognizing that CNAs are often the frontline caregivers for individuals living with dementia, we have focused on equipping them with the knowledge and skills needed to provide compassionate, evidence-based care. Through this initiative, we have trained hundreds of CNAs and fostered long-standing partnerships with key stakeholders, including nursing facilities, the Kentucky Office of Dementia Services, the Kentucky Cabinet for Health- Department of Behavioral Health, Development Intellectual Disabilities, and the Department of Public Health. These strong partnerships have not only expanded access to dementia care education but have also laid the foundation for sustainable workforce development initiatives. Building on this momentum, we are now developing a CNA apprenticeship program, designed to create a structured career pathway that integrates dementia training with hands-on mentorship and professional growth opportunities. This framework will help address Kentucky’s ongoing need for a well-trained, dementia-capable workforce by providing CNAs with continued education, career advancement opportunities, and long-term retention support. This paper session will highlight the evolution of our training initiatives, the impact of our collaborations, and the next steps in developing the CNA apprenticeship program. Participants will gain insights into effective strategies for workforce development, partnership-building, and the integration of dementia-specific training into CNA education, ensuring a skilled and sustainable caregiving workforce for Kentucky’s aging population.

